# Porcine epidemic diarrhea virus E protein causes endoplasmic reticulum stress and up-regulates interleukin-8 expression

**DOI:** 10.1186/1743-422X-10-26

**Published:** 2013-01-19

**Authors:** Xingang Xu, Honglei Zhang, Qi Zhang, Jie Dong, Yabing Liang, Yong Huang, Hung-Jen Liu, Dewen Tong

**Affiliations:** 1 College of Veterinary Medicine, Northwest A&F University, Yangling, Shaanxi, 712100, China; 2 Institute of Molecular Biology, National Chung Hsing University, Taichung, 402, Taiwan; 3 Agricultural Biotechnology Center, National Chung Hsing University, Taichung, 402, Taiwan

**Keywords:** PEDV, E protein, ER stress, IL-8, NF-Κb, Bcl-2

## Abstract

**Background:**

Porcine epidemic diarrhea virus (PEDV) is an important pathogen in swine and is responsible for substantial economic losses. Previous studies suggest that the PEDV E protein plays an important role in the viral assembly process. However, the subcellular localization and other functions of PEDV E protein still require more research.

**Methods:**

The subcellular localization and function of PEDV E protein were investigated by examining its effects on cell growth, cell cycle progression, interleukin-8 (IL-8) expression and cell survival.

**Results:**

The results show that plenty of PEDV E protein is localized in the ER, with small quantities localized in the nucleus. The PEDV E protein has no effect on the intestinal epithelial cells (IEC) growth, cell cycle and cyclin A expression. The cells expressing PEDV E protein express higher levels of IL-8 than control cells. Further studies show that PEDV E protein induced endoplasmic reticulum (ER) stress and activated NF-κB which is responsible for the up-regulation of IL-8 and Bcl-2 expression.

**Conclusions:**

This study shows that the PEDV E protein is localized in the ER and the nucleus and it can cause ER stress. The PEDV E protein had no effect on the IEC growth and cell cycle. In addition, the PEDV E protein is able to up-regulate IL-8 and Bcl-2 expression.

## Background

Porcine epidemic diarrhea (PED) is an acute and highly contagious enteric disease of swine characterized by severe enteritis, vomiting, and watery diarrhea and results in high mortality in piglets [1]. The causative agent belonging to the family Coronaviridae is porcine epidemic diarrhea virus (PEDV), which is first reported by Pensaert and DeBouck in Belgium and the United Kingdom [2,3]. PED is currently a source of concern in Asia countries, where outbreaks are often more acute and severe than those observed in Europe [4]. PED is one of the most important diseases incurring economic loss in many swine-raising countries, mainly due to its high prevalence, compared to the rare incidence of transmissible gastroenteritis (TGE) and the asymptomatic characteristics of the Rotavirus (RV) infections [3,5]. PEDV is an enveloped virus possessing an approximately 28 kb, positive-sense, single-stranded RNA genome with a 5’ cap and a 3’ polyadenylated tail [4,6]. The genome is comprised of seven open reading frames (ORFs) that encode four structural proteins and three non-structural proteins which are arranged on the genome in the order 5’-replicase (1a/1b)–S-ORF3–E–M–N–3’ [7-9]. Genes for the major structural proteins spike (S, 180–220 kDa), membrane (M, 27–32 kDa), nucleocapsid (N, 58 kDa) and small membrane (E, 7 kDa) are located downstream of the polymerase gene [10,11].

The E protein plays an important role during coronavirus budding and transiently resides in a pre-Golgi compartment before progressing to the Golgi apparatus. Studies on the assembly of coronavirus structural proteins by heterologous mammalian expression systems have shown that coexpression of E and M proteins from bovine coronavirus (BCoV), MHV, TGEV, IBV, and SARS-CoV results in the formation of virus like-particles (VLPs) that are morphologically identical to spikeless virions. Moreover, it has been determined that both the MHV and IBV E proteins are sufficient for the generation of VLPs [12]. Recently, a recombinant MHV virus was constructed with the E gene deleted. This virus replicates with a low infectious titer, suggesting that E protein is critical, but not essential for MHV replication in vitro [12]. Presently, no data were reported about the subcellular localization of PEDV E protein, its effects on cell growth and cell cycle progression. The porcine intestinal epithelial cell (IEC) line is the target cell of PEDV. The epithelial cells in the gut serve as a physical barrier, restricting the movement of components and the passage of potentially harmful microorganisms between the lumen and the underlying mucosa [13]. This study initiates the subcellular localization of PEDV E protein and elucidates the effects and mechanisms of this protein on cell growth and cell cycle.

The results show that the expression of PEDV E protein has no effect on cell growth, cell cycle and cyclin A expression in IEC. Furthermore, we show that plenty of PEDV E protein is localized in the ER and a little in the nucleus. PEDV E protein can induce ER stress. Under conditions of ER stress, the unfolded protein response (UPR) can initiate inflammation in mammalian cells and these responses are thought to be essential in the pathogenesis of inflammatory diseases [14,15]. IL-8 as a pro-inflammatory neutrophil chemotactic factor plays an important role in the promotion of cell survival signaling [16,17]. However, there is no report that PEDV E protein affects IL-8 expression in IEC. Our studies show that PEDV E protein up-regulates IL-8 expression which is associated with NF-κB activation. Moreover, the cells expressing PEDV E protein causes high expression of the anti-apoptotic protein Bcl-2. The experiment has potentially important implications for understanding the molecular mechanisms of PED pathogenesis. To our knowledge, this is the first report about PEDV E protein function on porcine intestinal epithelial cell.

## Results

### Construction of a recombinant plasmid pEGFP-N1-E and PEDV E protein expression

**Figure 1:**
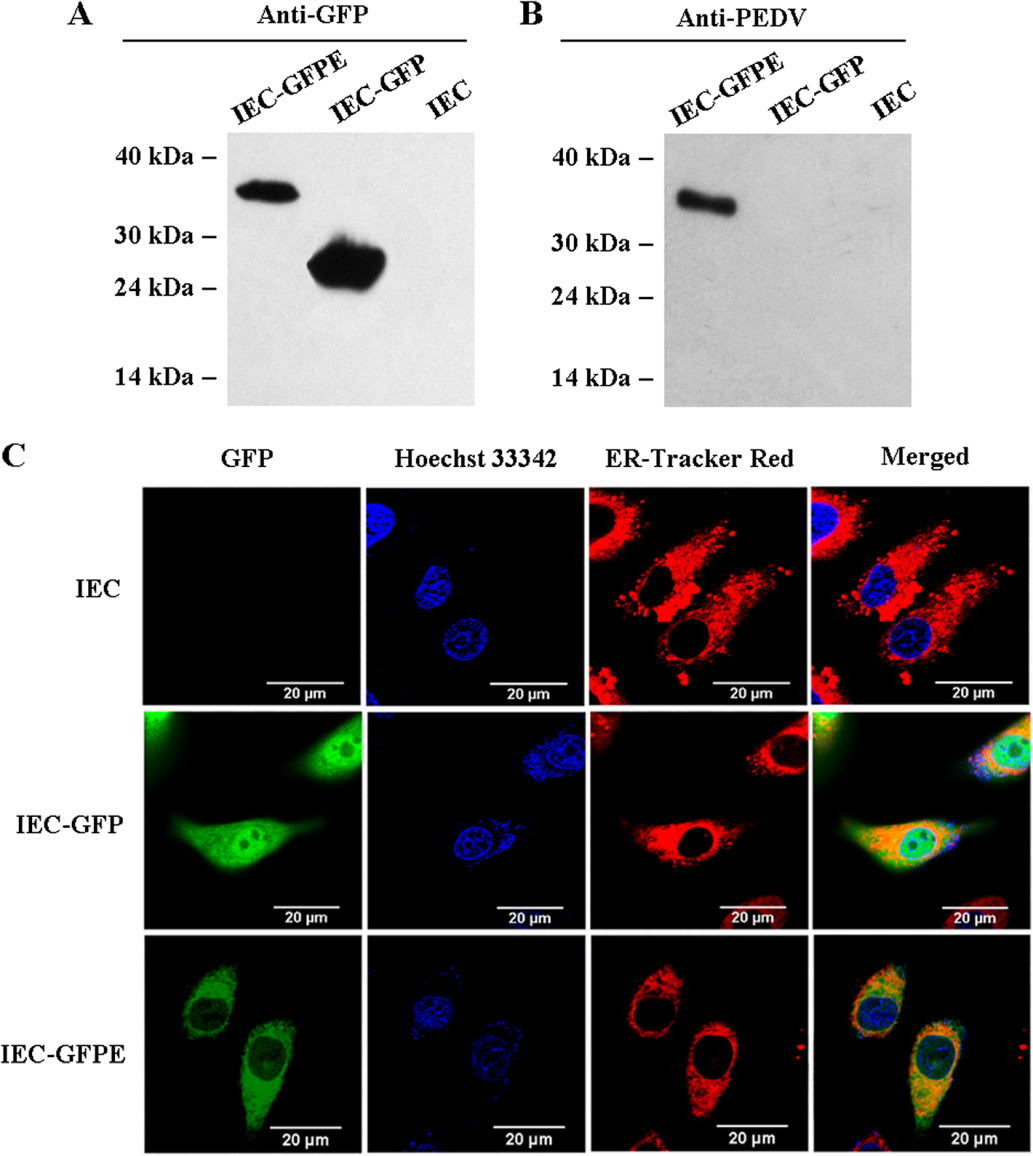
**Detection and subcellular localization of GFP-E fusion protein in IEC cells.** (**A**) Western blot analyses of expression products of GFP-E fusion protein in IEC cells. The cells were subjected to Western blot using anti-GFP antibody. Proteins were isolated from whole extracts of the stable cell lines expressing GFP-E protein and control cells. (**B**) The cells were subjected to Western blot using porcine anti-PEDV antibody. (**C**) Detection of GFP-E fusion protein subcellular localization in IEC cells by confocal microscopy. All the cell lines were stained by Hoechst33342 and ER-Tracker™ Red. Merged images showed co-localization of GFP-E protein primarily in the ER, with small amounts translocating in the nucleus. Bar = 20 μm for all the figures.

The Western blot assay (Figure 1A) showed that the cells transfected with the pEGFP-N1-E plasmid and pEGFP-N1 plasmid expressed approximate molecular mass of 35 kDa and 27 kDa proteins that were detected by anti-GFP monoclonal antibodies. At the same time, The Western blot assay (Figure 1B) showed that the cells transfected with the pEGFP-N1-E plasmid expressed an approximate molecular mass of 35 kDa protein that were detected by anti- PEDV polyclonal antibodies. Since the molecular mass of GFP is known to be approximate 27 kDa, this is in agreement with the predicted size of E protein approximate 8–9 kDa.

### PEDV E protein subcellular localization

The subcellular localization of E protein was investigated by confocal fluorescence microscopy. The results show that plenty of PEDV E protein was localized in the ER and a little in the nucleus while the control GFP protein was distributed throughout the whole cell (Figure 1C).

### PEDV E proteins have no effect on cell proliferation

**Figure 2:**
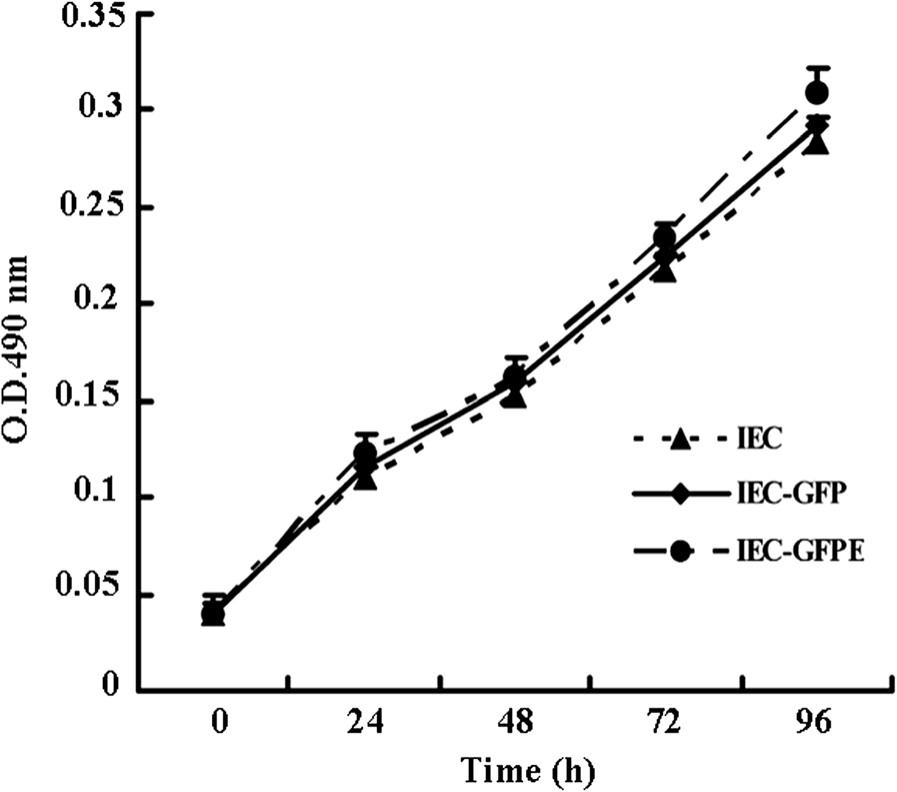
**Cell proliferation assays of the stable E protein expressing cells.** The MTT assay was used to measure proliferation of 3 × 10^3^ cells from IEC cell lines over time. Each data set represents the mean ± S.D. of six replicates.

Compared with the GFP protein expressing cells and the control cells, the E protein expressing cells show a slight change in mitotic activity which causes a little change cell number after a certain period. The results suggest that cell proliferation of E expressing cells did not change over a time course (Figure 2).

### PEDV E protein can not affect cell cycle

To investigate whether PEDV E protein expression has effect on cell cycle, flow cytometric analysis was performed based on DNA content in nuclei stained with PI. The proportions of G0/G1 phase, S-phase and G2/M phases for the control cells were 71.5%, 22.4% and 6.0%, respectively. For IEC expressing GFP, the proportions of the phases were G0/G1: 77%, S-phase: 16.4%, and G2/M: 6.5%, whereas for GFP-E-expressing IEC stable cells, the proportions were G0/G1: 74.4%, S-phase: 18.9% and G2/M: 6.7%. The histograms were quantitatively further analyzed to determine the percentage of cells in each of the G0/G1, S, and G2/M phases, where G0/G1 phase cells show a 2 N DNA content and G2/M phase cells show a 4 N DNA content. The results show that relative to control cells, PEDV E protein expression caused a slightly change in the proportion of cells in the each phase. Taken together, these results strongly suggest that PEDV E protein has no effect on cell cycle.

### PEDV E protein has no effect on cyclin A expression

One key regulator of cell cycle progression from the S phase to the G2/M phase is cyclin A. To understand the effect of E protein expression on cell cycle, we examined cyclin A protein levels in transfected cells and control cells using western blot assay. The result showed Cyclin A expression level was slightly changed in cells that expressed E protein compared with control cells. The result indicates that the expression of E had no change on cyclin A expression. To further support these findings, quantitative real-time RT-PCR was employed. The result shows that cyclin A mRNA levels in the GFP-E-expressing cells were also no change compared with control cells. This suggests that PEDV E protein has no effect on cyclin A protein expression.

### PEDV E causes ER stress via up-regulation of GRP78 and activation of NF-κB

**Figure 3:**
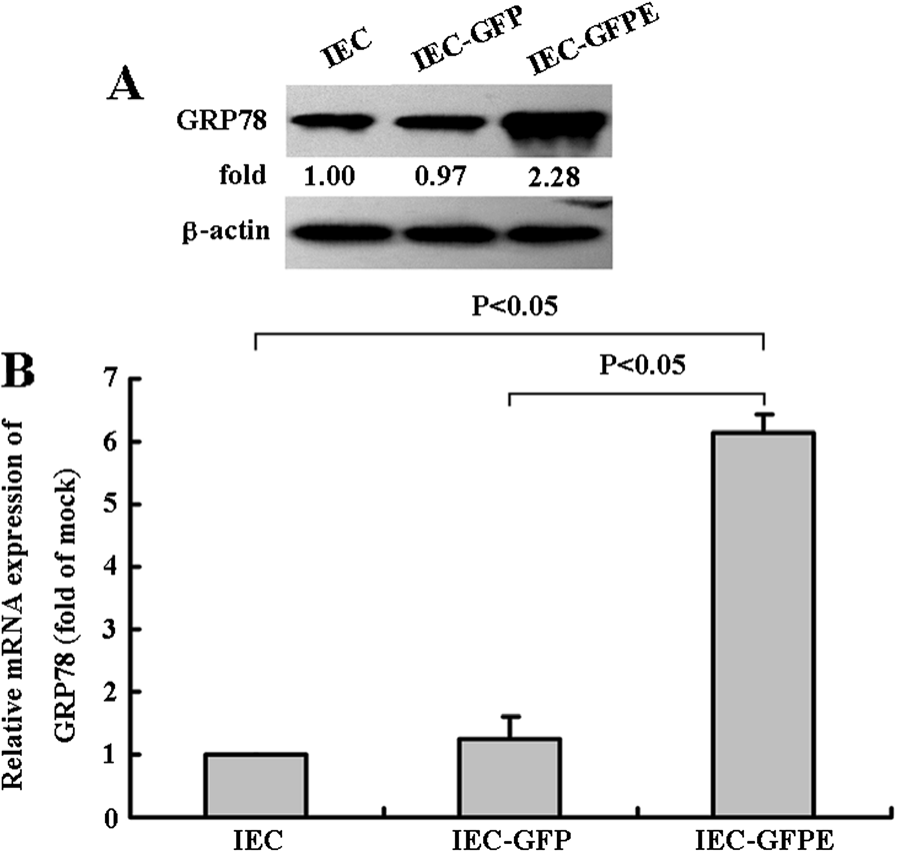
**Effect on porcine GRP78 expression by PEDV E expression in cultured IEC cells.** (**A**) The level of GRP78 expression was determined by western blot with the mouse monoclonal antibodies against GRP78 in all cell lines. β-actin was used as an internal loading control. (**B**) Total RNA was extracted from cells expressing either GFP alone, GFP-E or untransfected cells. Real-time PCR analysis of GRP78 mRNA levels were normalized to the corresponding CT value for porcine β-actin mRNA. The results are means ± SD and representative of three independent experiments.

To analyze the expression of glucose regulated protein 78 (GRP78), we chose a well characterized ER chaperone protein that is a marker of ER stress [18-20]. We examined the GRP78 protein levels in transfected cells and control cells using western blot assay. GRP78 expression level was significantly up-regulated in cell lines that expressed E protein compared with control cells (Figure 3A). Moreover, the GRP78 transcription level detected using real-time PCR assay was also significantly increased in GFP-N-expressing cell line compared with controls (Figure 3B).

**Figure 4:**
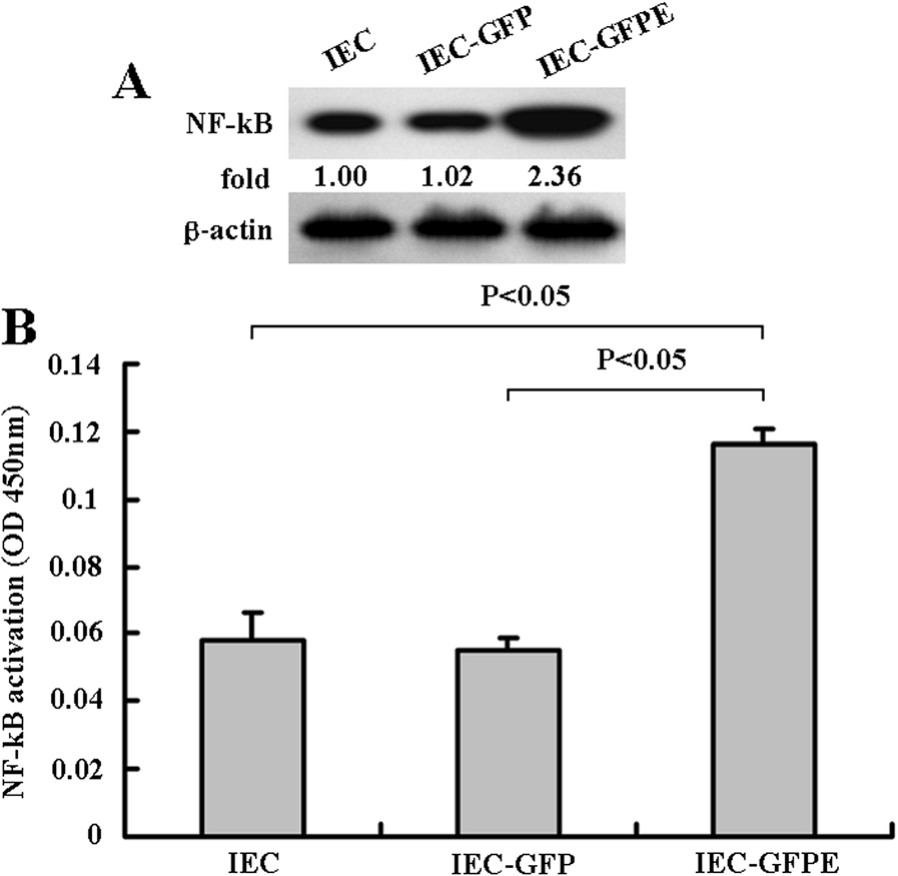
**PEDV E expression increases NF-κB activity in IEC cell lines.** (**A**) NF-κB p65 activation was determined by Western blot with the monoclonal antibodies against NF-κB p65 in all cell lines. β-actin was used as an internal loading control. (**B**) NF-κB p65 activation was determined using the TransAM assay. The data represent the mean and standard deviation from three different experiments.

Analysis of the activity of NF-κB in GFP-E-expressing cell lines demonstrates that the NF-κB was significantly activated compared with control cells (Figure 4A and B). Together, these analyses show that expression of PEDV E protein results in the up-regulation of GRP78 and NF-κB which suggests the E protein has a role in ER stress activation.

### PEDV E protein up-regulates IL-8 expression

**Figure 5:**
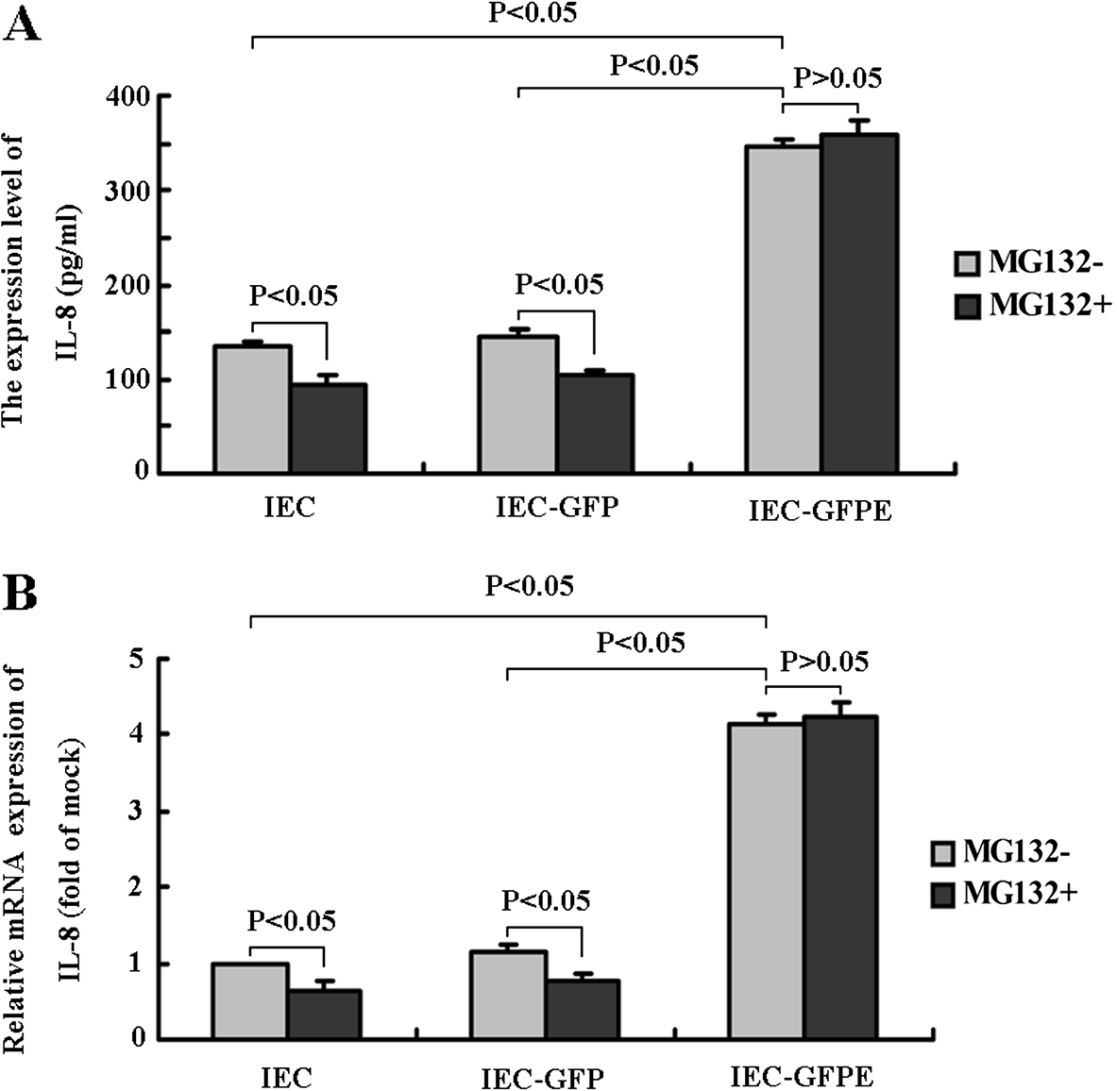
**PEDV E up-regulates IL-8 expression in IEC cells.** (**A**) The concentrations of IL-8 in GFP-E expressing IEC or control cells (treated or untreated with MG132) culture supernatants were measured by ELISA. Data are mean ± SD and representative of three independent experiments. (**B**) The effect of PEDV E expression on porcine IL-8 transcription in cultured IEC cells. Total RNA was extracted from cells expressing either GFP alone, GFP-E fusion, or untransfected cells. Real-time RT-PCR analysis of IL-8 mRNA levels were normalized to the corresponding CT value for porcine β-actin mRNA. The results are means ± SD and representative of three independent experiments.

The secretion of IL-8 in the supernatant of the untransfected and transfected cells was examined using ELISA assay. As shown in Figure 5A, GFP-E expressing cells were found to express higher levels of IL-8 compared to control GFP expressing cells and the untransfected cells. After the treatment with MG132, the level of IL-8 in the supernatants from control cells was significantly decreased. In contrast, there was no change in supernatants from the GFP-E expressing cells. An investigation of the transcriptional levels of IL-8 using real-time quantitative PCR found that the mRNA levels of IL-8 in the GFP-E expressing cells is also higher than in the GFP transfected cells and untransfected cells (Figure 5B). These results suggested that PEDV E up-regulates IL-8 expression in IECs. IL-8 expression is regulated by activation of NF-κB and, in turn, the activation of NF-κB is associated with ER stress during the viral infection [21,22]. The results suggest that PEDV E expression results in ER stress and NF-κB activation which is responsible for the up-regulation of IL-8.

### PEDV E protein up-regulates Bcl-2 expression

**Figure 6:**
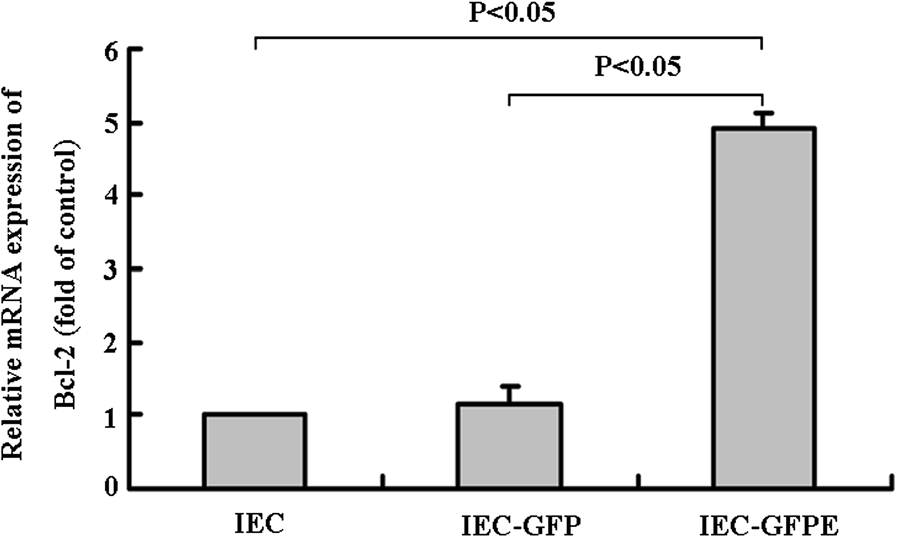
**Effect on porcine Bcl-2 expression by PEDV E protein in cultured IEC cells.** Real-time PCR analysis of Bcl-2 mRNA levels were normalized to the corresponding CT value for porcine β-actin mRNA. Data are means ± SD from three independent experiments.

The anti-apoptotic molecule Bcl-2 is tightly regulated by the transcription factor NF-κb [23]. Also, Bcl-2 is associated with cell survival [24-26]. A quantitative real-time RT-PCR was employed and the results show that Bcl-2 expression in the cells of GFP-E protein expression is higher than in the control cells (Figure 6). The results suggest that PEDV E protein may play a very important role in protecting the host cells from functional damage or apoptosis.

## Discussion

Recent years, many studies were focus on the gene sequence analysis of PEDV, and genetic and phylogenetic analysis based on the S, M and ORF3 genes have been used to determine the relatedness of PEDV isolates [11,27-29]. However, the subcellular localization and function of PEDV E protein is still unclear. Also, the function of this protein is yet to be studied, particularly with regard to its effect on host cell physiological changes. In this study, we constructed a eukaryotic expression vector and generated stably expressing cell lines of PEDV E in fusion with the GFP protein that allowed analysis of its properties. Co-localization studies clearly show that plenty of PEDV E protein is localized in the ER with small amounts collecting in the nucleus. In this study, Western blot analysis revealed that cyclin A protein levels in the cells expressing PEDV E protein were slightly change compared with control cells, suggesting that PEDV E protein had no effect on host cell proliferation and cell cycle.

Our observations show that the PEDV E protein is likely to be responsible for inducing ER stress. In this study, the results show that PEDV E protein was localized in the ER and the nucleus and able to induce ER stress, as indicated by the significant up-regulation of the molecule chaperon GRP78, a typical marker of ER stress.

The ER has essential roles in multiple cellular processes that are required for normal cellular functions and cell survival [30]. Viruses use the ER as an integral part of their replication strategy; they must contend with the ER stress response and the downstream consequences of ER stress signalling, including the initiation of an inflammatory response through the activation of NF-κB [14,15,22]. IL-8 as a pro-inflammatory neutrophil chemotactic factor plays an important role in the promotion of cell survival signaling and antagonizes the anti-viral activities of interferon. In this study, the results show that PEDV E protein was able to up-regulate IL-8 expression in host cells. PEDV E protein induced ER stress and significantly activated NF-κB which consequently caused the promotion of IL-8 expression. Further research suggests that IL-8 expression in control cells treated with MG132 was significantly decreased compared with untreated cells. However, the IL-8 production from the cells of GFP-E protein expression treated with MG132 was not significantly changed compared with the untreated cells. These results show that MG132 was able to inhibit IL-8 expression in control cells and the E protein was able to antagonize MG132 function.

In addition, NF-κB is a transcription factor that controls the expression of a variety of genes involved, not only in innate and adaptive immunity, but also in cell survival [31-33]. As we know, Bcl-2 as an anti-apoptotic molecule which is associated with cell survival [24-26]. Furthermore, Bcl-2 expression is regulated by the NF-κB [23]. In this study, the anti-apoptotic molecule Bcl-2 was significantly elevated in the cells expressing PEDV E protein. The results show that PEDV E protein may play an important role in protecting the host cells from morphological and functional damage or apoptosis.

## Conclusions

In conclusion, the bulk of PEDV E protein localizes in the ER with trace amounts in the nucleus. PEDV E protein has no effect on the IEC growth, cell cycle and cyclin A expression. The PEDV E protein is able to up-regulate IL-8 expression in IEC. The up-regulation of IL-8 and Bcl-2 expression is ascribed to the ER stress response and activation of NF-κB which are induced by PEDV E protein. Thus, the data suggest that PEDV E protein likely plays an important role in the inflammatory response and the persistent PEDV infection. This study provides novel findings for the function of the PEDV E protein which are likely to be very useful for understanding the molecular mechanisms of PEDV pathogenesis.

## Methods

### Vectors, plasmids and cells

The pEGFP-N1 eukaryotic expression vector was purchased from Clontech (USA) and *Escherichia coli* DH5α used for cloning were purchased from Tiangen Biotech (China). In this study, the PEDV Shaanxi strain was isolated from intestinal tract contents of PEDV infected piglets in Shaanxi Province of China and E gene of PEDV was amplified as described previously [34]. The established swine intestinal epithelial cells (IEC) which were kindly provided by Prof. Yan-Ming Zhang, College of Veterinary Medicine, Northwest A&F University, were cultured as described previously [35]. Briefly, IEC cells were grown in Dulbecco’s modified eagle medium (DMEM) (Gibco BRL, Gaithersburg, MD, US) supplemented with 10% heat-inactivated new born calf serum (Gibco BRL, Gaithersburg, MD, US), 100 IU of penicillin and 100 μg of streptomycin per ml, at 37°C in a 5% CO2 atmosphere incubator. The culture medium was replaced every 3 days.

### Antibodies and reagents

Mouse monoclonal antibodies against cyclin A, GRP78, NF-κB p65, β-actin were purchased from Santa Cruz Biotechnology (Santa Cruz, Inc., CA, US). Porcine anti-PEDV polyclonal antibody was kindly provided by China Animal Health and Epidemiology Center (Qingdao, China). Mouse anti-GFP monoclonal antibody was purchased from Millipore (USA), Horseradish peroxidase (HRP)-conjugated secondary antibody was purchased from Pierce (Pierce, Rockford, IL, US). The MG132 proteasome inhibitor was purchased from Calbiochem (USA) and the nuclear staining dye Hoechst33342 and ER-Tracker™ Red probe were obtained from Invitrogen (USA).

### Construction of recombinant plasmid and transfection

The primers used to amplify E gene of PEDV were as follows: forward primer (PEDV-XhoI), 5’-CCGCTCGAGATGCTACAATTAGTGAATGATA-3’ (25444–25465 of CV777 strain) and reverse primer (PEDV- EcoRI), 5’-CCGGAATTCCTACGTCAATAACAGTACTGGG G-3’ (25650–25671 of CV777 strain). The restriction sites are underlined. The primers were designed according to the archived PEDV CV777 strain nucleotide sequence (GenBank: AF353511.1) and synthesized by Shanghai Invitrogen (China), while the primers were used for the PCR amplification and were designed with 5’ terminal restriction enzyme recognition sites for aid cloning into pEGFP-N1. The PCR product was detected by 1.0% agarose gel electrophoresis, purified from the gel and digested with restriction enzymes to be cloned into the pEGFP-N1 expression vector. The recombinant plasmid was named as pEGFP-E and recovered from transformed E. coli using a plasmid mini-kit (Axygen, China) and identified by enzyme digestion and DNA sequencing.

IEC cells were seeded into 6-well dishes 24 h before being transfected (up to 70-80% confluence). Cells were transfected with pEGFP-E and pEGFP-N1 control vector using Lipofectamine 2000 (Invitrogen, USA) and maintained (up to 80-90% confluence) in selection media containing 1200 μg/mL G418 for two weeks. When all control cells had evidence of death in the presence of the selection agents, cultures transfected with pEGFP-E and pEGFP-N1 were propagated for two further weeks in medium containing 600 μg/mL G418. The resulting stably transfected cell lines expressing either GFP or GFP-E fusion proteins were used for subsequent analysis.

### Confocal microscopy

To examine the expression and subcellular localization of PEDV E protein, the stable cell lines expressing GFP-E protein or control cells (GFP and untransfected cells) were grown on glass bottom dishes (35 mm) and washed with Hank’s balanced salt solution (HBSS) and incubated with Hoechst33342 at 37°C for 10 min, and then washed twice with HBSS. Cells were then incubated with ER-Tracker Red probe (Invitrogen, USA) at 37°C for 25 min and washed with HBSS for twice. Images were viewed by laser confocal scanning microscopy (Model LSM510 META, Zeiss, Germany).

### Western blot analysis

Cells were harvested and washed with ice-cold PBS, then treated with ice-cold RIPA lysis buffer with 1 mM phenylmethyl sulfonylfluoride (PMSF). Cell lysates were centrifuged at 12 000 × g at 4°C for 10 min. Protein concentrations were measured using BCA Protein Assay Reagent (Pierce, Rockford, IL, US). Equivalent amounts of proteins were loaded and electrophoresed on 8-12% sodium dodecyl sulfate polyacrylamide gel electrophoresis (SDS-PAGE). Subsequently, proteins were transferred to polyvinylidene difluoride (PVDF) membranes (Millipore Corp, Atlanta,GA, US). The membranes were blocked with 5% nonfat dry milk at room temperature for 1 h, and then incubated with indicated primary antibodies over night at 4°C, followed by HRP-conjugated secondary antibodies at room temperature for 1 h. The signal was detected by enhanced chemiluminescence (ECL) reagents (Pierce, Rockford, IL, US).

### Cell proliferation assay

The MTT cell proliferation assay was performed to determine the growth properties of PEDV E-expressing and control cells according to the manufacturer’s instructions. Briefly, cells were seeded in 96-well culture plates at a concentration of 2 × 10^3^ cells per well in 200 μL culture medium. After incubation at 37°C with 5% CO_2_ for 24 h, 48 h, 72 h and 96 h, the culture medium was carefully replaced with 200 μL of a fresh medium without disturbing the cells. Twenty microlitres of 3-(4,5-dimethylthiazol-2-yl) 2,5-diphenylte-trazolium bromide (MTT, 5 mg/ml) (Sigma, St. Louis, USA) reagent was added to each well and incubated in a CO_2_ incubator at 37°C for 4 h. After 4 h incubation, the reactions were stopped by addition of 100 μL of DMSO into each well. The absorbance at a wavelength of 490 nm was read on a microplate reader (Model 680, Bio-Rad, USA) at appropriate time intervals. The experiments were independently repeated three times.

### Cell cycle analysis by flow cytometry

The cell cycle was measured by using propidium iodide staining. Briefly, approximately 2 × 10^6^ cells of the stable cell lines and control cells were treated with trypsin, washed with phosphate-buffered saline (PBS) for twice, resuspended in 75% ethanol and fixed at 4°C for 3 days. Cells were washed with PBS and resuspended in PBS containing 20 μg/mL of RNase A and 50 μg/mL of propidium iodide (PI) and incubated at 4°C for 30 min in the dark. Finally, the nuclear DNA content was determined by a Coulter Epics XL flow cytometer (Beckman Coulter, USA).

### Real-time quantitative PCR analysis

Total RNA was extracted from cells using Trizol reagent (Invitrogen, California, USA) according to the manufacturer’s instructions and reverse transcribed with M-MLV reverse transcriptase, oligo(dT)18 primers and 2 μg of total RNA. The expression of genes was quantified using Bio-Rad iQ5 Real Time PCR System by means of a real-time quantitative PCR assay (qRT-PCR). The primers for qRT-PCR in this study were shown in Table 1. Reactions were carried out in 25 μl volume containing SYBR Premix Ex TaqTM II (Takara, Dalian, China), sense and anti-sense primers (0.4 μM) and target cDNA (4 ng). The cycling conditions were 95°C for 5 min, followed by 40 cycles of 95°C for 5 s, 60°C for 30 s. A negative control was included in each run and the specificity of amplification reaction was checked by melting curve (Tm value) analysis. The individual samples were normalized for genome equivalents using the respective CT value for the porcine β-actin housekeeping gene. The relative quantification of gene expression was analyzed by the two-ddCt method as described previously [36].

**Table 1:** Sequences of primer pairs used for qRT-PCR

**Gene**	**Forward primer (5’-3’)**	**Reverse primer (5’-3’)**	**Product(bp)**	**Accession no.**
Cyclin A	AAGTTTGATAGATGCTGACCCGTAC	GCTGTGGTGCTCTGAGGTAGGT	194	GQ265874
GRP78	AATGGCCGTGTGGAGATCA	GAGCTGGTTCTTGGCTGCAT	114	X92446
IL-8	CTGGCTGTTGCCTTCTTG	TCGTGGAATGCGTATTTATG	113	M86923
Bcl-2	TTGTGGCCTTCTTTGAGTTCG	CTACCCAGCCTCCGTTATCC	150	XM_003121700.1
β-actin	GGACTTCGAGCAGGAGATGG	AGGAAGGAGGGCTGGAAGAG	138	XM_003124280.1

### Detection of NF-κB activity

To determine the alteration of NF-κB activity by GFP and GFP-E proteins in the established cell lines, the level of NF-κB activity was measured using western blot assay and the NF-κB p65 TransAM kit (Active Motif) according to the manufacturer’s instructions. Briefly, cells nuclear extraction was prepared by using the Nuclear Extract Kit (KeyGEN, Nanjing, China) and protein concentrations were measured using the BCA Protein Assay Reagent (Pierce, Rockford, IL, US). Lysates (50 μg total proteins) were incubated in ELISA wells coated with the oligo-nucleotide motif recognized by active p65, then detected using a specific antibody against p65, followed by a horseradish peroxidase (HRP)-conjugated secondary antibody. The colorimetric reaction was measured at 450 nm. This experiment was repeated three times.

### Enzyme-linked immunosorbent assay (ELISA)

The stable PEDV E gene expressing cells and the control cells were seeded in 24-well plates at a density of 1 × 10^5^ cells/ml in DMEM with 10% new born calf serum (NCS) and cultured for 48 h. In some experiments, MG132 previously found to block IL-8 expression was added after 24 h [37]. The culture medium was then collected and centrifuged in a microcentrifuge at 1, 000 × g for 5 min to remove debris, the supernatants were then frozen at −80°C until analysed. The concentrations of IL-8 were measured using a swine IL-8 ELISA kit according to the manufacturer’s instructions (Invitrogen, USA).

### Statistical analysis

Data are shown as the means ± SD of three independent experiments done in triplicate. For each assay, student’s t-test was used for statistical comparison. A value of *P* < 0.05 was considered significant.

## Competing interests

The authors declare that they have no conflicts of interest.

## Authors’ contributions

XX and HZ performed the majority of experiments and involved in manuscript preparation, QZ, JD, YL and YH participated part of the experiments. DT and HJL conceived of the study, participate in its design and coordination, and revised the manuscript. All authors read and approved the final manuscript.

## Authors’ information

Dr. De-Wen Tong, professor of College of Veterinary Medicine, Northwest A&F University, Vice Dean of College of Veterinary Medicine, Northwest A&F University. Dr. Hung-Jen Liu, professor of Institute of Molecular Biology, National Chung Hsing University. Dr. Xin-Gang Xu and Dr. Yong Huang are associate professors of College of Veterinary Medicine, Northwest A&F University. Honglei Zhang, graduate students of College of Veterinary Medicine, Northwest A&F University.
